# Advances in drug delivery applications of modified bacterial cellulose-based materials

**DOI:** 10.3389/fbioe.2023.1252706

**Published:** 2023-08-04

**Authors:** Shuya Liang

**Affiliations:** Department of Dermatology, Affiliated Hospital of Qingdao University, Qingdao, Shandong, China

**Keywords:** bacterial cellulose, modification, biomedical application, biomaterials, drug delivery

## Abstract

Bacterial cellulose (BC) is generated by certain species of bacteria and comprises polysaccharides with unique physical, chemical, and mechanical characteristics. Due to its outstanding biocompatibility, high purity, excellent mechanical strength, high water absorption, and highly porous structure, bacterial cellulose has been recently investigated for biomedical application. However, the pure form of bacterial cellulose is hardly used as a biomedical material due to some of its inherent shortcomings. To extend its applications in drug delivery, modifications of native bacterial cellulose are widely used to improve its properties. Usually, bacterial cellulose modifications can be carried out by physical, chemical, and biological methods. In this review, a brief introduction to bacterial cellulose and its production and fabrication is first given, followed by up-to-date and in-depth discussions of modification. Finally, we focus on the potential applications of bacterial cellulose as a drug delivery system.

## 1 Introduction

In recent years, cellulose, one of the most abundant naturally occurring polymers on Earth, has been extensively explored for different areas of biomedical application due to its renewable, sustainable, biodegradable, and biocompatible properties (Moniri et al., 2017; Portela et al., 2019; Deng et al., 2022). Chemically, cellulose is a linear homopolysaccharide polymer of β-D-glucopyranose units covalently linked together by β-glycosidic bonds (Dong et al., 2012; Portela et al., 2019). Plant cellulose usually associates with hemicellulose, pectin, lignin, arabinose, and other biogenic compounds, which makes it hard to obtain highly pure cellulose (Avcioglu, 2022). Apart from being frequently obtained from the plant primary cell wall, cellulose is also discovered to be produced by various microorganisms such as algae (Han et al., 2022), fungi (Klemm et al., 2005), and some bacteria (Lin et al., 2020; Arserim-Uçar et al., 2021). Bacterial cellulose (BC), with characteristic microstructures and produced by certain bacterial species, has gained widespread interest due to its outstanding biological and physicochemical properties over plant cellulose including high purity, better crystallinity, excellent water-holding capacity, high mechanical strength, ultrafine network nature, and mouldability *in situ* (Rajwade et al., 2015; Lahiri et al., 2021; Aditya et al., 2022; Popa et al., 2022). Additionally, BC can be synthesized in different forms depending on various fermentation processes, including fibrils, sphere-like particles, pellicles, and tubes, to be a potential material for various applications (Qiu and Netravali, 2014; Mbituyimana et al., 2021).

The human body, an amazing system, is made up of complex tissues, which is prone to diseases. Various drugs are introduced into the human body to attain positive therapeutic effects, which involve varied delivery systems. The choice of material as a primary focus of drug delivery is unquestionably important because it further determines the most appropriate route of administration and results in the ultimate objective of therapeutic success (Mensah et al., 2021). The drug molecules can be directly delivered to the target site or administrated by systemic circulation (Coelho et al., 2010). Oral administration and injection have been the main drug delivery routes for a long time. However, long-standing problems of conventional administration methods, such as accurate dosing, time duration, targeted delivery, and tunable release rate, pose challenges in applications. In order to achieve this, the now-called drug delivery systems, including nanoparticles, liposomes, microemulsions, and niosomes, have been developed to increase the bioavailability (Vishali et al., 2019; Geng et al., 2021a; Geng et al., 2021b; Souto et al., 2022). Over the past few decades, BC-based drug delivery systems have attracted considerable attention as ideal candidates due to their remarkable biocompatibility, biodegradability, and nontoxicity. Furthermore, the existence of a great number of hydroxyl (OH) groups on the BC surface provides abundant binding sites for physiochemical and mechanical modifications (Pan et al., 2023).

**FIGURE 1:**
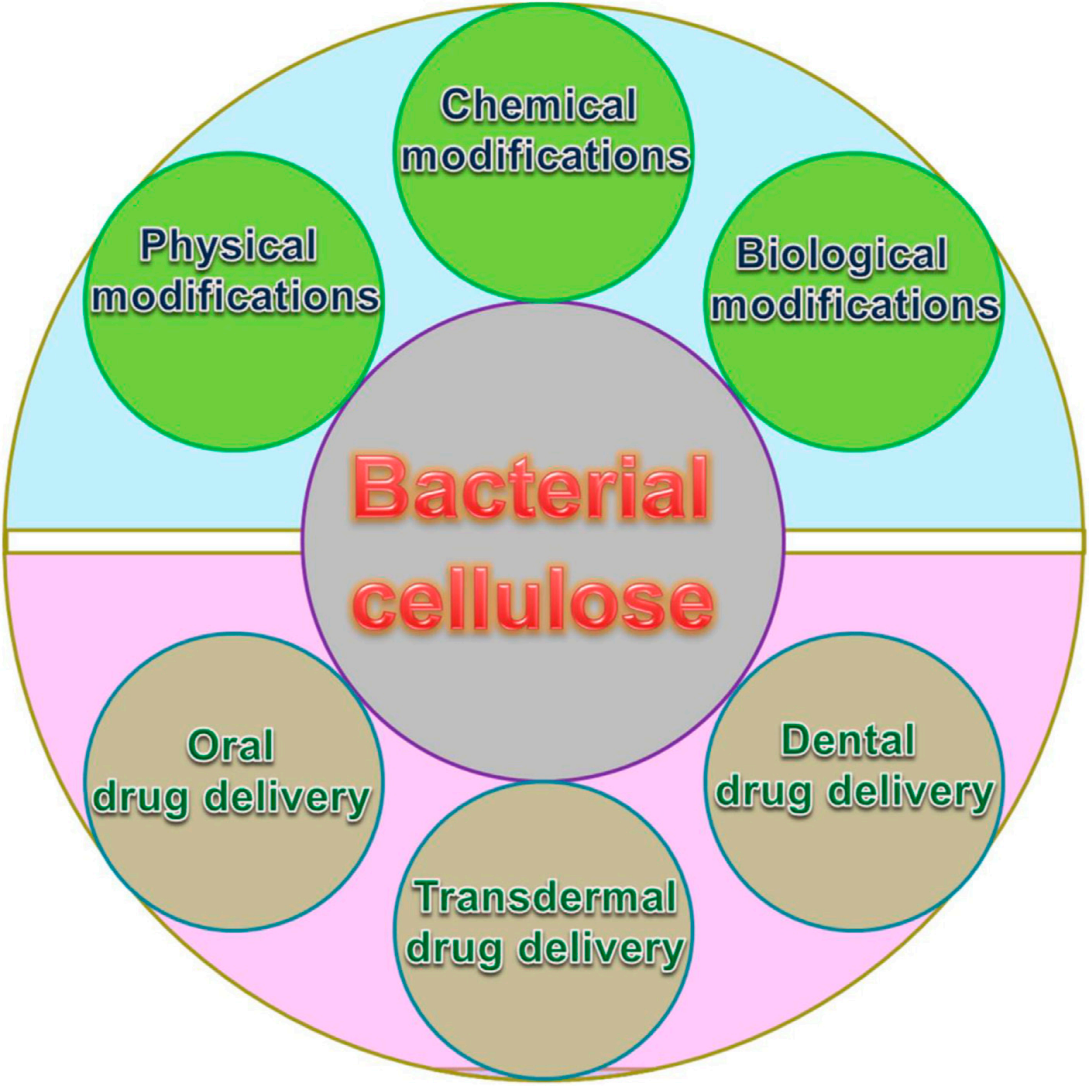
Schematic representation of the modification and drug delivery of BC.

In this article, the latest developments of the understanding of BC-based biomaterials and the studies on their fabrication and BC-based drug delivery are summarized and highlighted. We briefly introduce the production and fabrication of BC, followed by a summary of its applications in drug delivery (as shown in Figure 1). Finally, we conclude the progress in fascinating and challenging fields.

## 2 Production and fabrication of BC

### 2.1 Biosynthesis of BC

The British scientist Brown first discovered BC on the surface of vinegar fermentation broth in 1886 as a kind of cellulose material generated from *Acetobacter xylinum* (Brown, 1886). Until now, Gram-negative bacterial species, including *Gluconacetobacter*, *Achromobacter*, *Agrobacterium, Pseudomonas*, *Rhizobium*, *Sarcina*, *Komagataeibacter*, *Salmonella*, and *Alcaligenes* can also be employed to synthesize BC (Gregory et al., 2021; Mbituyimana et al., 2021). In general, the synthesis mechanism of BC involves four steps: 1) glucose is phosphorylated by means of the conversion of enzyme glucokinase to glucose-6-phosphate (Glc-6-P); 2) Glc-6-P is isomerized by phosphoglucomutase into glucose-1-phosphate (Glc-1-P); 3) Glc-1-P is converted into uridine diphosphate glucose (UDPG) by UDPG pyrophosphorylase; 4) finally, UDPG is synthesized by cellulose synthase into cellulose (Liu et al., 2020; Souza et al., 2020). During biosynthesis, BC molecules are first produced in the bacterial cell and then secreted into the outside medium across small pores of the cell outer membrane (Yun et al., 2010). Normally, approximately 10–15 glucan chains will be associated with each other to arrange sub-fibrils of approximately 1.5 nm in width (Chen et al., 2021). Subsequently, these fibrils aggregate side-by-side to form 30–80-nm-wide mature cellulose nanofiber, which is stabilized by intra- and intermolecular hydrogen bonds. According to the different fermentation processes, BC can be obtained in various structures such as aster-like/sphere-like hydrogels produced under agitated/shaking culture conditions and thin-film hydrogels synthesized under static culture conditions (Wang et al., 2019; Fooladi et al., 2023). Consequently, BC has been considered a versatile candidate for drug delivery systems owing to its unique structure and properties.

### 2.2 Modifications of BC

Although BC displays many beneficial properties, it is an unsuitable biomaterial for controlled drug delivery due to the failure to resist the freedom of movement of small molecules in the highly porous structure of BC (Mohammadi et al., 2023). Therefore, modification of BC has become inevitable to develop its properties for desired application in drug delivery. Such modifications will improve the physical–mechanical and surface characteristics of BC. Currently, a number of modification techniques have been developed applying functional polymers, biosynthetic methods, and solvents to fabricate various BC-based biomaterials with superior physicochemical properties (Badshah et al., 2018; Chen et al., 2022). A number of the advantages and disadvantages of three BC modification methods are presented in Table 1. Overall, there are many recent studies on physical, chemical, and biological modifications of BC, which will be summarized in the following sections.

**TABLE 1:** Comparison of physical, chemical, and biological modifications of BC.

Modification method	Advantage	Disadvantage	References
**Physical modification**	Synthesis means are simple and gentle (adsorption, ultrasonication, and coating)	Chemical structure of BC is maintained; applications of BC are decided by added materials	Qian et al. (2023)
**Chemical modification**	Easily introduced functional groups into the BC structure by esterification, etherification, grafting, or oxidation	Complex operation process; expensive approach; tedious and delicate reaction procedures	Gao et al. (2019)
			Singhsa et al. (2018)
**Biological modification**	Easy processing	Suspended particles with quick penetration lead to difficult composite synthesis	Andriani et al. (2020)
	Multiple synthesis methods	Limited applications of BC via the agitation culture method	Shavyrkina et al. (2021)
		Basic structure of BC is disturbed	Luo et al. (2020)

#### 2.2.1 Physical modifications

Physical modifications are the most widely used methods to endow BC with unique responsive properties from other materials due to their simple principle and easy operation. Coating can keep the initial morphology of underlying materials without incurring damage. Organic polymers and inorganic materials are usually used as surface coating to protect BC from external corrosion (including strong oxidants, acids, and bases) (Huang et al., 2022). Xi et al. have investigated the physical and chemical characteristics of poly(lactide-co-glycolide) (PLGA) or polydopamine (PDA) as an innovative coating material for BC (Lai et al., 2019). The results show that PDA increases the mechanical strength of the BC membrane, while the PLGA-coated BC membrane improves cell proliferation and collagen accumulation *in vivo*. Lim et al. have successfully prepared new hydroxyapatite-encapsulated BC scaffolds for bone–tissue engineering (Ahn et al., 2015). New bone formation was observed to be significantly increased by hydroxyapatite-coated BC scaffolds in a rat calvarian defect model compared with those from BC scaffolds. In addition to being used as a supporting material, BC can also be utilized as a precursor to prepare water-soluble polyethylene glycol (PEG)-doped BC as a drug delivery platform. Beekmann et al. proposed to dope PEG in the BC structure to improve the transparency of the substrate (Beekmann et al., 2020). The addition of PEG enlarges the pore size and increases water absorption capacity, leading to enhanced drug loading and release capacity. Direct mixing is the simple method for producing BC-based functional composites. Wei et al. fabricated a BC-reinforced dual-cross-linked hydrogel through the freeze–thaw process (Huang et al., 2020). The mixture possesses both the electrical property of conductive materials and mechanical characteristic of BC, which exhibits excellent mechanical strength and electrical signal response. Kareem et al. treated BC nanofibrils with gamma irradiation for altering their crystallinity index and size (Hamed et al., 2022). The structure of BC nanofibrils was not changed after treatment, while the formation of lamellae, cross-linking, and recrystallization of BC nanofibrils enhanced the potential application of BC. Paradee et al. fabricated pectin/BC hydrogel composites for transdermal drug delivery (Krathumkhet et al., 2021). In this study, BC was successfully incorporated into a ppy/pectin/BC hydrogel composite, resulting in improved mechanical properties. Although physical modifications of BC are simple to fabricate and accurately control the structure of materials, it is difficult to satisfy the demands of mass preparation of material because of the high cost and requirement of complex instruments.

#### 2.2.2 Chemical modifications

Due to the existence of many OH groups on the surface of BC, chemical modifications can be achieved between the BC substrate and active materials via chemical reactions. The high degree of polymerization and crystallinity index cause the difficult dissolution of BC, resulting in chemical modifications mainly taking place in amorphous regions and glucopyranoside (Lei et al., 2020). Many chemical reactions, including esterification, oxidation, acetylation, and graft copolymerization, are still utilized for improving BC characteristics (Qian et al., 2023). Esterification of BC is an acylation process of OH groups employing carboxylic acid as an acylating agent (Qian et al., 2023). Ngai et al. described a simple and low-pollution surface-esterification strategy for fabricating BC/Kombucha tea-based hydrophobic BC films (Jiang et al., 2023). The strategy uses alkenyl succinic anhydrides with various chain lengths as esterifying agents to introduce pendant carboxylic groups and provide multi-cross-linking sites for strengthening the BC structure, resulting in endowing the BC film with significant resistance to water, UV, oxygen, and bacteria. Sufiandi et al. selected citric acid (CA) and polyethylene glycol 600 (PEG-600) as the cross-linking agent and plasticizer, respectively, to apply into the BC film through the immersion method (Pratama et al., 2022). CA increases the formation of covalent bonding to OH groups on either BC or PEG-600 through esterification, leading to an improvement in both tensile strength and tensile modulus.

**FIGURE 2:**
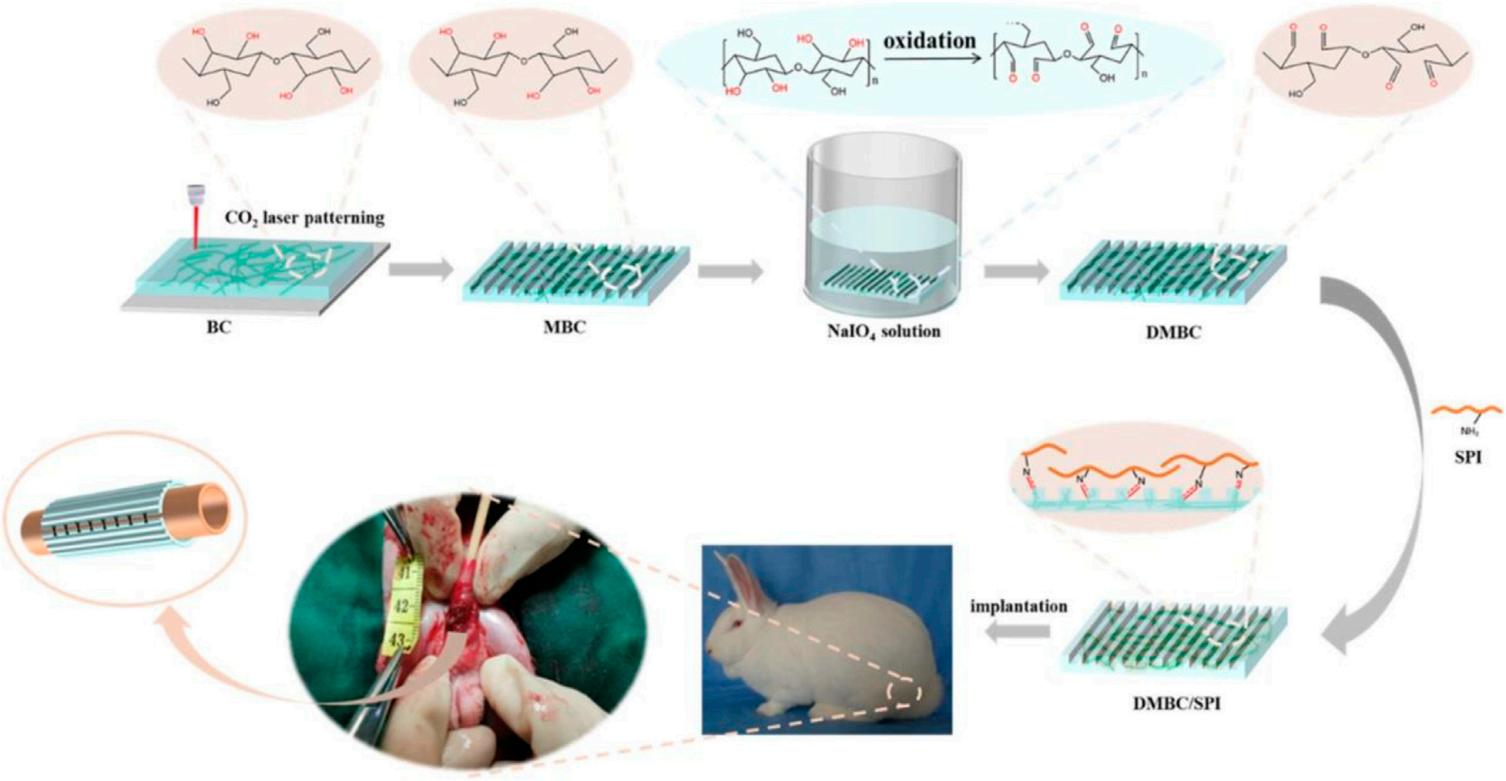
Preparation of the double-modified BC/soybean protein isolate. Reproduced with permission from Yang et al. (2022). Copyright 2021, American Chemical Society.

Oxidation is a commonly used method to introduce carboxylic acid or aldehyde functional groups on the surface of BC (Zhou et al., 2021). Changes in the physical and chemical properties of BC can be induced according to the relating oxidizing conditions, including pH value, reaction time, temperature, and oxidizing agents (Solomevich et al., 2020). Although many oxidizing agents, including persulfates, permanganates, hydrogen peroxide, phosphoric acids, and nitrogen dioxide, have been developed to oxidize the OH groups to carbonyl or carboxyl groups in BC, 2,2,6,6-tetramethylpiperidine-1-oxyl (TEMPO) is still generally utilized to oxidize BC (Solomevich et al., 2020). Lee et al. used TEMPO to oxidize primary OH groups on bacterial cellulose nanofiber (BCNF) to carboxylate groups for conjugating involucrin antibody onto the BCNF backbone to synthesize a BCNF-based bioadhesive (Kim et al., 2022). The present process has the main advantage that it has an insignificant effect on the crystallinity and mechanical strength of BCNF. Similarly, Feng et al. developed a new high-performance carboxyl-functionalized BC-based ionogel based on TEMPO-oxidized BC and ionic liquid using the cosolvent volatilization method (Fan et al., 2022). The *in situ* oxidation of BC shows minimum damage at the fiber, and as-fabricated carboxyl-functionalized ionogels with excellent tensile strength, high adhesion, and good biodegradability are obtained. Nitrogen dioxide is another widely used oxidizing agent, which selectively converts the primary OH group of BC to the carboxyl group (Wahid et al., 2021). The oxidation of BC by nitrogen dioxide can obtain a higher number of carboxylic groups, leading to the excellent adsorption capacity of the drug molecule. Yurkshtovich et al. obtained an amount of carboxyl groups increasing to 19.5% by using an HNO_3_/H_3_PO_4_-NaNO_2_ mixture for the oxidation of BC, while BC had a significantly lower oxidation degree in other oxidation systems (Solomevich et al., 2021). More importantly, cephalexin-loaded oxidized BC with prolonged drug release properties demonstrates high antibacterial activity. Sodium periodate, another strong oxidizing agent, is ionized to periodate ions in aqueous conditions to react with BC by electrostatic attraction, oxidizing the vicinal groups at the positions C2 and C3 in cellulose units, forming 2,3-dialdehyde cellulose (Gomes Luz et al., 2022). Cai et al. prepared a double-modified BC/soybean protein isolate (DMBC/SPI) utilized as a urethral tissue engineering scaffold through laser hole formation and periodate-induced selective oxidation (Yang et al., 2022). The introduction of highly reactive dialdehyde is beneficial for SPI compounding on BC to increase the biocompatibility of DMBC/SPI (Figure 2).

**FIGURE 3:**
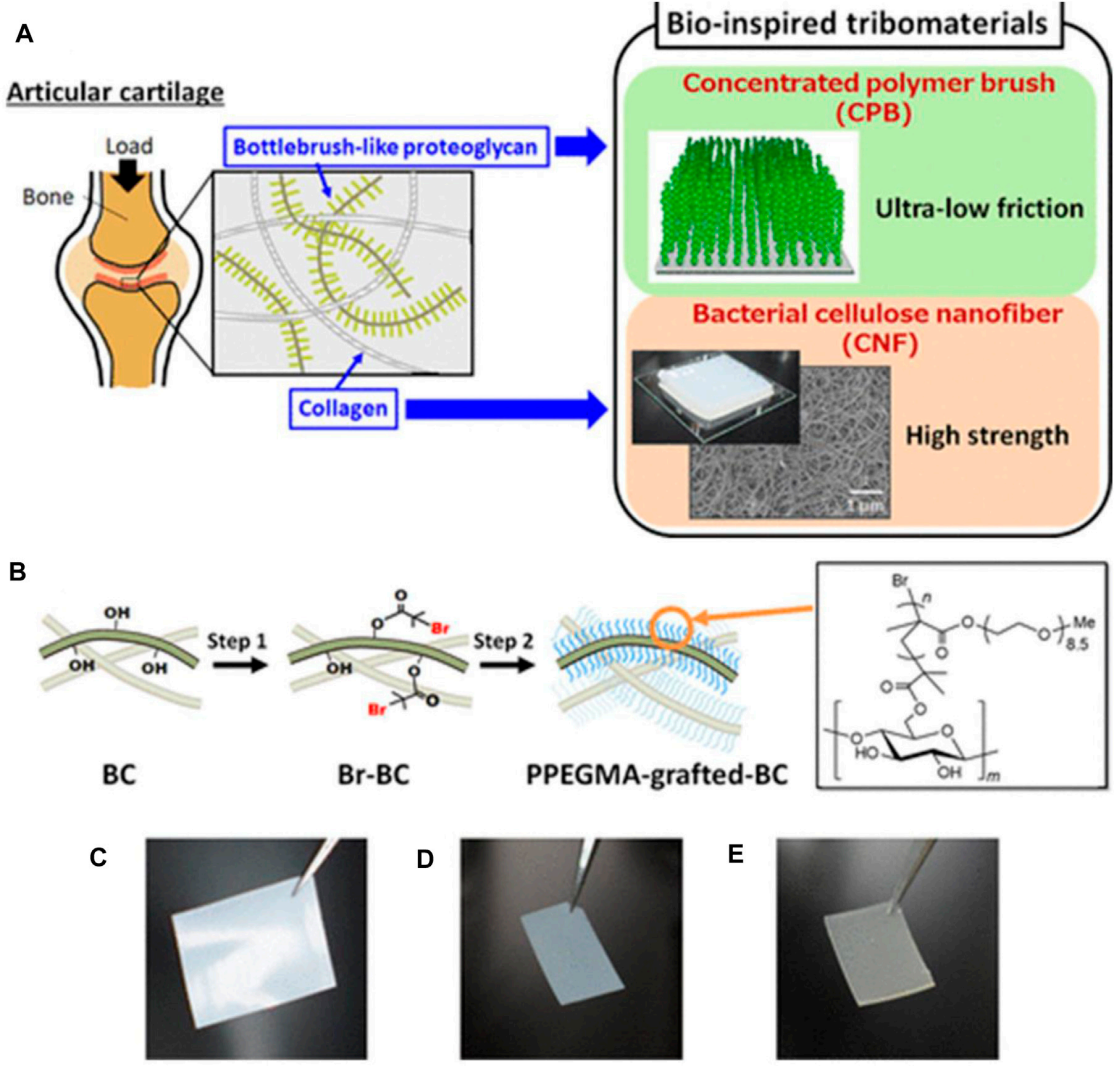
**(A)** Schematic of the bioinspired tribomaterials combining the polymer brush and BC nanofiber. **(B)** Two-step synthesis of poly[poly(ethylene glycol) methyl ether methacrylate] (PPEGMA)-grafted BC films. **(C–E)** Representative diagrams of pristine BC **(C)**, initiator-immobilized BC film **(D)**, and poly[poly(ethylene glycol) methyl ether methacrylate]-grafted BC films in a dry state **(E)**. Reproduced with permission from Sakakibara et al. (2021). Copyright 2021, American Chemical Society.

**FIGURE 4:**
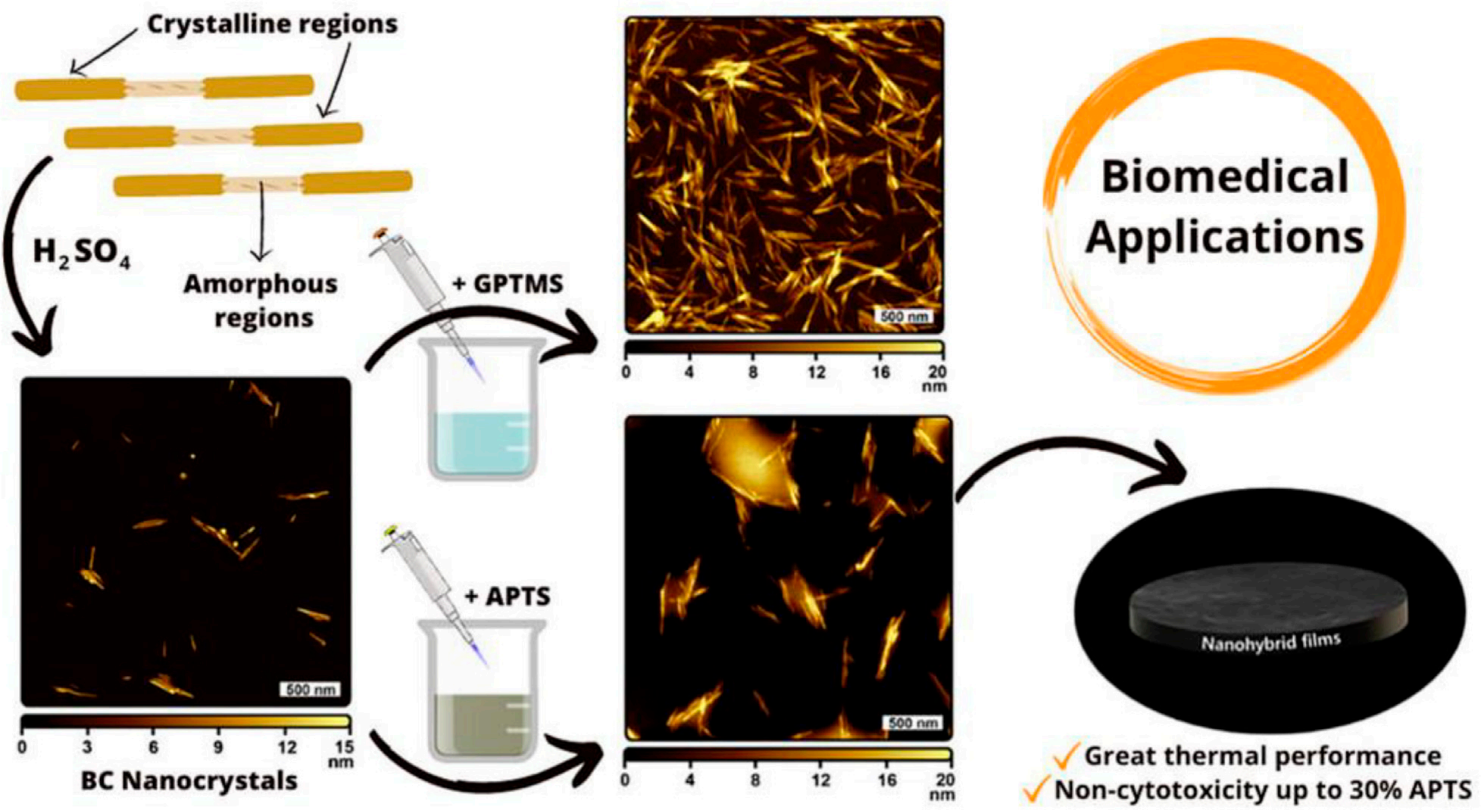
Isolation of BC nanocrystals from the BC dressing residue and applications. Reproduced with permission from Lima et al. (2022). Copyright 2022, American Chemical Society.

On the other hand, sulfation is a commonly used approach for synthesizing BC-based functional composites. Cellulose sulfate can be formed through sulfation in sulfuric acid or an SO_3_–pyridine complex, with several properties such as anticoagulating, antivirus, and antibacterial activity (Song et al., 2021). Sun et al. prepared sulfated BC to create a composite with gelatin as the building block of cell scaffolds (Li et al., 2023). The results show that the sulfation of BC renders composite scaffolds with good cytocompatibility, hemocompatibility, and anticoagulant effects. Moreover, the high sulfation degree of BC can stimulate fibroblast cell growth with a growth factor and be used to encapsulate cells and enzymes for blood contacting biomaterials (Peschel et al., 2010). In addition to the aforementioned ways, chemical modification of BC also includes etherification and grafting. Carboxymethyl BC is a cellulose ether, which is prepared in a two-step process: first, BC is activated by alkali solution treatment and then reacted with monochloroacetic acid or sodium monochloroacetate by etherification. For instance, Lin et al. prepared carboxymethyl BC with different substitution degrees. BC is immersed in sodium hydroxide/ethanol and then reacted with sodium chloroacetate (Lin et al., 2015). The protein adsorption behavior of the resultant membrane shows the sorption quantity of bovine serum albumin relying on the degree of substitution. Grafting also plays a significant role in chemical modification for endowing new properties to BC. The commonly used methods of BC modification are surface-induced atom transfer radical polymerization (ATRP), conventional synthetic method, and cross-linking of silane coupling agents (Kumar et al., 2021; Sriplai and Pinitsoontorn, 2021; Peng et al., 2023). Poly[poly(ethylene glycol) methyl ether methacrylate] molecules are attached to the surface of the BC nanofiber by surface-initiated ATRP, obtaining excellent mechanical properties similar to those of human articular cartilage (Figure 3) (Sakakibara et al., 2021). Silane coupling agent cross-linking of BC relates to the use of cross-linking agents, including glutaraldehyde and N,N′-methylene bisacrylamide, to attach BC with other polymers to prepare 3D-network structure products for drug-controlled release (Zhang et al., 2021). Barud et al. reported a new approach for creating surface-modified bacterial cellulose nanocrystals (BCNCs) using 3-glycidyloxypropyltrimethoxysilane (GPTMS) or 3-aminopropyltriethoxysilane (APTES) (Lima et al., 2022). The structural, morphological, thermal, and cytotoxicological properties of siloxane-coated BCNC are elucidated, aiming to pave sustainable avenues toward drug delivery systems and scaffolds (as displayed in Figure 4). Ying et al. first used amino-functionalized BC as an enzyme carrier to immobilize horseradish peroxidase by glutaraldehyde coupling (Yu et al., 2019). The results show that glutaraldehyde-based cross-linking of BC increases the binding activity and reusability of the enzyme protein.

#### 2.2.3 Modifications via microbial fermentation

BC can be modified in the bacterial culture process by adding other materials into the medium or altering the available carbon source or controlling the culture condition (He et al., 2021). The composition, pore size, and pore structures of BC can be regulated with different factors, providing BC with special characteristics. Moreover, the fermentation medium plays a vital role in BC production in the process of microbial culture. Various additional molecules or units are added into culture media, which enter the microbial metabolic pathway as carbon sources; thus, the physical and chemical features of BC are effectively regulated (Li et al., 2015). Many biological modifications of BC have been beneficially explored over the past 20 years. Liu et al. reported the effects of aggregation-induced emission-based photosensitizer-modified glucose (TPEPy-Glc) contained in the culture medium on the surface of BC (Liu et al., 2022). The direct biosynthesis method endows BC with remarkable fluorescence brightness and light-triggered photodynamic antibacterial activity. Zhao et al. found that the activity of carboxylic acid moieties increased by adding D-saccharic acid potassium salt as a carbon source into the bacterial culture medium (Zeng et al., 2023). Gunduz et al. indicated that the addition of hyaluronic acid/gelatin (HA/Gel) into the medium contributes to modified-BC scaffold production by an *in situ* fermentation process (Unal et al., 2021). The introduction of HA/Gel endows the BC composite scaffold with moderately hydrophilic property, which has great influence in cell adhesion and proliferation. Jiang et al. successfully fabricated a sodium alginate-BC (SA-BC) nanocomposite hydrogel via an *in situ* biosynthesis modification method (Ji et al., 2021). The enzymatic hydrolysate of glycerol-pretreated Moso bamboo is applied as a glucose-substitute carbon source in the culture medium to generate SA-BC, resulting in the formation of a highly porous net-like structure and enhanced thermal stability. Mahmoudi Rad et al. introduced green tea as a substrate to prepare BC/silver nanocomposites via a green approach with excellent antibacterial properties (Fadakar Sarkandi et al., 2021). To achieve this design, the silver nanoparticles are prepared by an *in situ* synthesis, along with BC production in the fermentation. Moreover, the excellent antibacterial results indicate the potential of the one-step facile green approach that can be widely used as a candidate for biomedical application. Although modifications via microbial fermentation can modify the physical, chemical, and microbiological characteristics of BC, some limitations remain. For instance, some additives cause a harmful effect during fermentation, thus endowing BC with undesirable properties. Therefore, more carefully conceived comparative research studies are necessary for further understanding the mechanisms of modification via microbial fermentation.

## 3 Applications of BC in drug delivery

Nowadays, BC-based biomaterials are extensively applied for drug delivery (Carvalho et al., 2019). Different methodologies have been carried out to investigate BC-based drug delivery systems, from superficial skin infections to cancer therapy. Furthermore, various drugs, including diclofenac, doxorubicin, ibuprofen, and tetracycline, have been successfully loaded into BC due to facile loading, simple usage, and similar drug release properties for drug delivery (Picheth et al., 2017). BC-based drug carriers have been applied in various drug delivery systems including oral drug delivery, transdermal drug delivery, dental drug delivery, and other BC-based drug delivery.

### 3.1 Oral drug delivery

**FIGURE 5:**
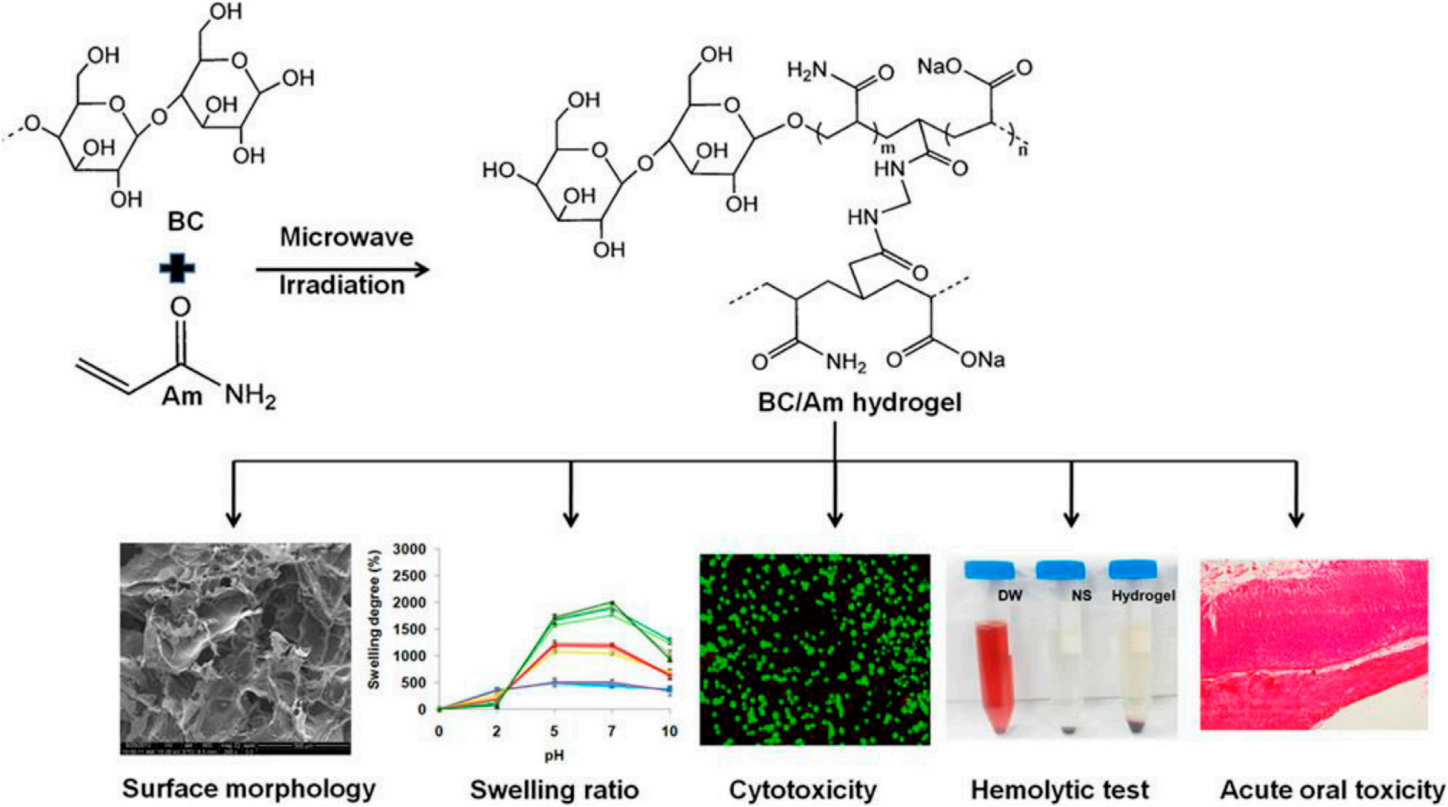
Preparation scheme of BC-based hydrogel synthesis, characterization, and toxicity studies of obtained hydrogels. Reproduced with permission from Pandey et al. (2014). Copyright 2014, American Chemical Society.

To date, BC as a versatile biomaterial has been applied for wound healing and artificial skin and tissue engineering (Ullah et al., 2017). Moreover, the Food and Drug Administration has described BC as generally recognized as safe, which confirms its safety and suitability in the development of oral drug delivery systems. Khan et al. prepared and evaluated single excipient-based BC matrices for oral drug delivery using famotidine and tizanidine as model drugs (Badshah et al., 2017). The studies reveal the chemical and thermal stability of drug-loaded BC-based matrices. Furthermore, compared to conventional tablets, the hydrated, or partially hydrated, or freeze-dried BC matrices show superior drug delivery characteristics. Retegi et al. biosynthesized BC/graphene oxide sphere-like hydrogels via an *in situ* route with an accurate control of their conformation for oral delivery (Urbina et al., 2020). The obtained results show that the graphene oxide concentration during BC biosynthesis determines the swelling capacity of spherical hydrogels. Additionally, the potential application of the as-prepared spherical hydrogels as nanocarriers was evaluated by carrying out the loading and release of ibuprofen in simulated intestinal fluid. Xin et al. investigated the latent capacity of a pH-responsive BC-g-poly(acrylic acid-co-acrylamide) hydrogel for oral sustained release drug delivery (Mohd Amin et al., 2014). The resultant hydrogels were successfully prepared by microwave-assisted copolymerization of the monomers to BC in a NaOH/urea mixture. The swelling behavior of pH-responsive hydrogels is evaluated in simulated gastric and intestinal fluid, suggesting that these BC-based hydrogels may be appropriate for an oral drug-controlled release carrier. Amin et al. synthesized and evaluated the safety and biocompatibility of BC/acrylamide (AM) pH-sensitive smart hydrogels as oral drug delivery vehicles (Figure 5) (Pandey et al., 2014). The hydrogels are prepared via a microwave irradiation method using N,N′ methylenebis (acrylamide) as a cross-linker, with solubilized BC in NaOH/urea solution. Theophylline was used as a model drug to study drug loading and release behaviors with varying BC concentrations. Furthermore, the cytotoxicity and acute gastrointestinal toxicity were studied for evaluating the biological safety of the prepared hydrogels as oral drug delivery carriers, and the results indicate the noncytotoxic nature of the BC-based hydrogel (Pandey et al., 2016). More recently, He et al. used the one-pot synthesis method to prepare a series of zwitterionic hydrogels using chitosan and BC as raw materials for oral drug release (Jiang et al., 2022). Partially oxidized BC and chitosan are used for the *in situ* formation of BC-AA-CS composite hydrogels via Schiff’s base reaction with enhanced mechanical properties and pH-responsiveness. In addition, the BC addition induces the porous structure to help BC-AA-CS maintain the desired drug release rates. Furthermore, due to the destabilization and degradation of proteinaceous drugs, they normally require frequent intravenous injections, leading to patient poor adherence. Oral proteinaceous drug delivery is a tremendous challenge and opportunity in modern medicine. BC-based materials as carriers show great promise in improving the pharmacodynamics and pharmacokinetics of proteins.

### 3.2 Transdermal drug delivery

**FIGURE 6:**
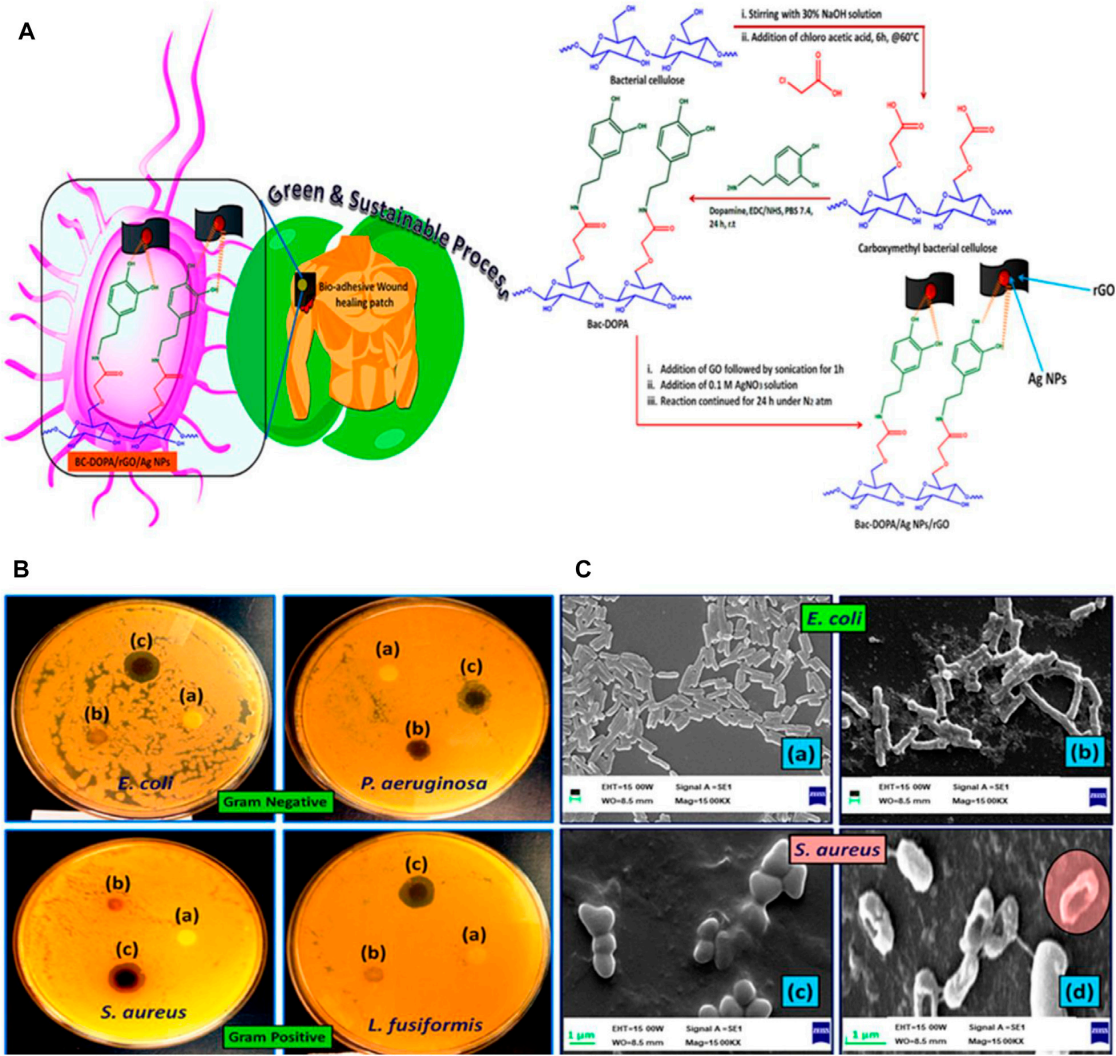
**(A)** Schematic of the modification of BC with dopamine. **(B)** Antimicrobial activity study of a BC-based complex against Gram-negative and Gram-positive bacteria. **(C)** Morphological analysis of the bacteria before and after treatment BC-DOPA/reduced graphene oxide/silver nanoparticle composite film patch. Reproduced with permission from Khamrai et al. (2019a). Copyright 2019, American Chemical Society.

Transdermal drug delivery presents a promising alternative for both oral administration and hypodermic administration (Ullah et al., 2016). Since ancient periods, people have applied various components on their skin for treatment, and in the present time, some transdermal methods have been designed for delivering drug molecules to systemic circulation. Due to the unique physical and mechanical characteristics, BC has attracted substantial interest for its potential in the transdermal drug delivery system. Klimowicz et al. considered the potential of BC membranes as a carrier containing ibuprofen and its amino acid ester salts for the therapeutic feasibility in transdermal drug delivery (Ossowicz-Rupniewska et al., 2021). *In vitro* studies show that the incorporation of L-valine isopropyl ester ibuprofenate or L-leucine isopropyl ester ibuprofenate in the surface of BC presents higher permeation rates than those obtained with ibuprofen, indicating the great potential of BC films used for transdermal application. Using calcium chloride as a physical cross-linking agent, Jantrawut et al. prepared BC-based hydrogels with alginate and/or pectin via ionic interaction for optimal hydrogel formulation with good antibacterial activities, used in transdermal drug delivery (Chanabodeechalermrung et al., 2022). Guan et al. developed a multifunctional theranostic platform employing magnetic hydrogel nanoparticles and BC for enhanced cancer targeting via transdermal drug delivery (Zhang et al., 2019). In this study, Fe_3_O_4_ nanoparticles were used as the core encapsulated with the hydrogel, loading doxorubicin and hematoporphyrin monomethyl ether, while the folic acid molecules graft on the structure of hydrogels, followed by the composites being encapsulated with the BC membrane. The results indicate that the synthesized BC/hydrogels are delivered transdermally using a magnetic field and laser for solving the limited penetration depth in breast cancer treatment. Kang et al. fabricated a semi-dissolving microneedle patch in a one-step process without drug loading by introducing TEMPO-oxidized BC nanofibers for effective transdermal drug delivery (Song et al., 2020). These two-layer patches fabricate TEMPO-oxidized BC nanofibers as an insoluble backing layer and the hydrophilic biopolymer mixture as a water-soluble needle layer by micro-molding. In particular, the TEMPO-oxidized BC nanofibers become highly dispersed in the aqueous solution, while maintaining their physical fiber structure and displaying excellent water absorption capacity. Kundu et al. synthesized a biocompatible gelatin-based hydrogel transdermal drug delivery patch containing polyelectrolyte-modified BC as a reinforcing agent (Khamrai et al., 2019b). The results indicate that the ionically functionalized self-assembled BC presents a sturdy cage for the gelatin molecule and modifies the ionic interlocking system in a pH 7.4 buffer solution after being damaged, for self-healing. Sáha et al. developed magnetic hybrid hydrogels, which involve alginate, casein, and BC impregnated with magnetic nanoparticles as potential injectable hydrogels for the transdermal drug delivery system (Patwa et al., 2020). These multicomponent hydrogels are prepared via 1-ethyl-3-(3-dimethylaminopropyl)-carbodiimide/N-hydroxy-succinimide interaction of alginate and casein and the supramolecular interaction of BC impregnated with iron nanoparticles. In addition, the incorporation of BC in alginate–casein hydrogels improves its mechanical properties and increases porosity and then promotes cell viability and adhesion. Kundu et al. prepared a novel mussel mimetic transdermal patch using a green resource, BC, derived from *Gluconacetobacter xylinus* for wound-healing applications (Khamrai et al., 2019a). To endow the mussel property, a catechol-holding compound is applied to chemically functionalize BC by an amidation reaction to endow an adhesive nature to the prepared transdermal patch (as shown in Figure 6). Then, the free OH group of dopamine-modified BC (BC-DOPA) was utilized to prepare the BC-DOPA/reduced graphene oxide/silver nanoparticle composite film. The biocompatibility and mechanical properties of the as-prepared composite transdermal patch indicate that mussel mimetic BC-based transdermal patches have a promising application in wound-healing therapy. Briefly, transdermal routes can be used as an entrance gate for BC into the drug delivery system, which facilitates drug delivery to the specific site. Although BC-based transdermal drug delivery platforms have yielded satisfactory *in vitro* and *in vivo* results, BC-based drug-loaded carriers on the market are still few. More issues relevant to the clinical application, including large-scale drug production, long-term storage, and drug-sustained/controlled release, should be studied.

### 3.3 Dental drug delivery

**FIGURE 7:**
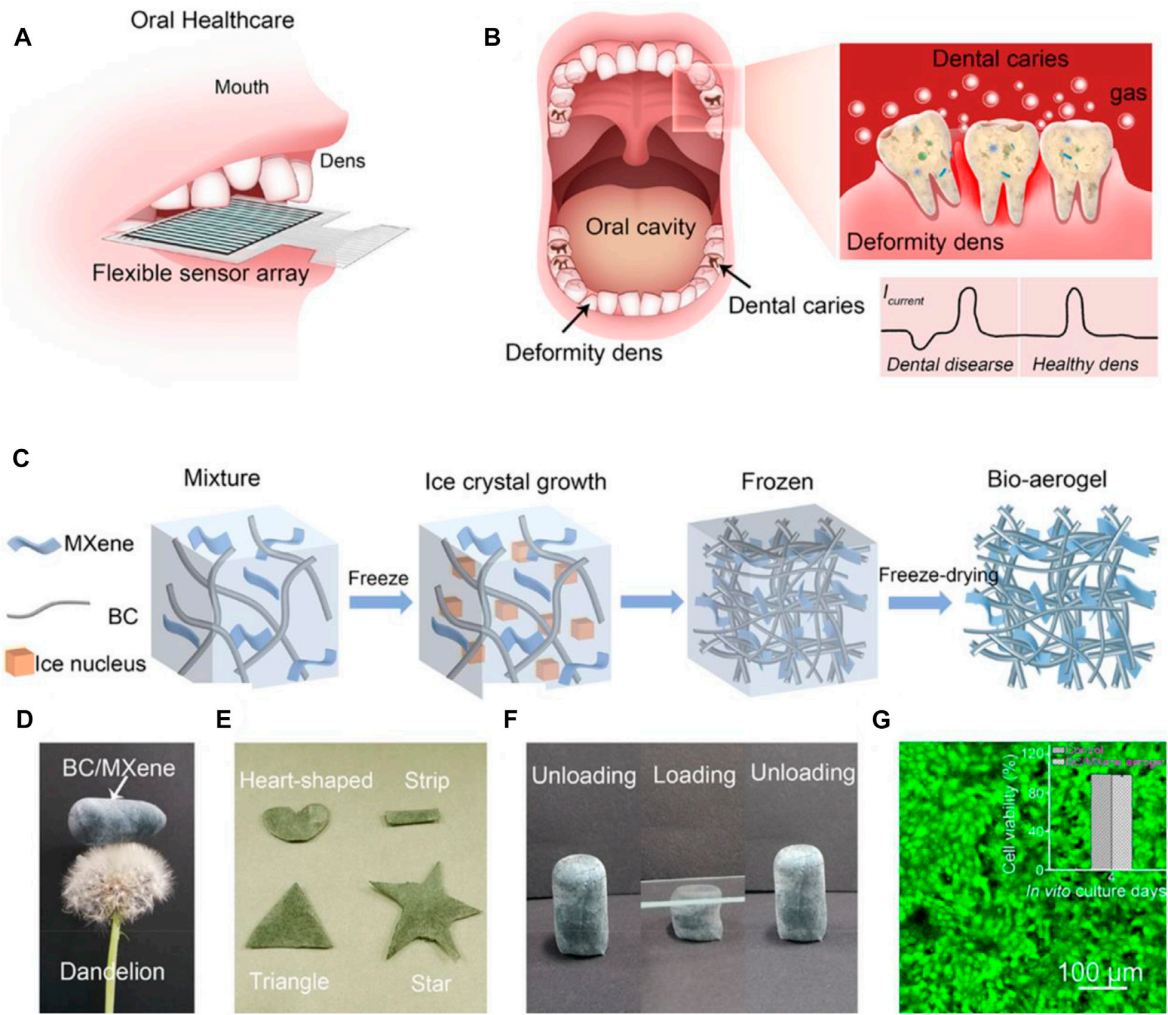
**(A)** and **(B)** Principles and illustration of the BC/MXene bioaerogel-based flexible platform. **(C)** Preparation schematic of the BC/MXene bioaerogel. **(D)** and **(E)** Representative images of the BC/MXene bioaerogel with lightweight and various shapes. **(F)** Representative images of the BC/MXene bioaerogel with unloading–loading state. **(G)** Representative fluorescent images of human immortalized epidermal cells cultured with the BC/MXene bioaerogel. The inset presents the viability of the cell over 4 days. Reproduced with permission from Jin et al. (2021). Copyright 2021, American Chemical Society.

Dental diseases, for example, caries and periodontal disease, are among the most common diseases in human beings (Nguyen et al., 2011). Different drug dosage forms have been designed to prevent and treat oral diseases, with toothpastes and mouthwashes being most popular. The drawback of conventional ways is the short residence time in the oral cavities, caused by salivary secretion, intermittent swallowing, food intake, and abrasion induced by soft tissue mobilization (Khezri et al., 2018). Prolonged drug release in the mouth shows great advantages in treating oral diseases. Using BC as a novel dental drug delivery system helps overcome this problem. This paper describes several BC-based biomedical applications that display BC as an ideal biomaterial for dental drug delivery. For example, BC has displayed great potential in wound dressing application and drug delivery due to its unique inherent properties. Kralisch et al. successfully combined both applications to investigate the application in dental therapy (Weyell et al., 2019). In order to achieve partial biodegradability under oral physiological environments while retaining its drug delivery property, the modified degradation behavior of BC by periodate oxidation was investigated. *In vitro* toxicity tests and agar diffusion tests show that the biocompatibility of periodate oxidation BC can be confirmed, whereas the antibiotic efficiency loaded with doxycycline hyclate against pathogenic oral bacteria is provided. This study indicates that the development of tailor-made BC-based biomaterials for dental applications is promising. Meier et al. developed bioabsorbable and bactericide membranes using oxidation methods for treating periodontal diseases caused by bacteria (Inoue et al., 2020). In this article, NaIO_4_ is used as the oxidizing agent to oxidize BC for adequate degradation time and suitable manipulation properties. Chlorhexidine was chosen as the drug model to investigate the drug release profile from the complexes of chlorhexidine: β-cyclodextrin. It was found that a strong chemical interaction occurred between chlorhexidine and oxidized-BC, contributing to significant retention of drugs, which is important for a prolonged antibacterial effect. Recently, Shen et al. reported a multifunctional and flexible BC/Ti_3_C_2_T_x_ MXene bioaerogel for monitoring occlusal force and early detection of dental diseases (Jin et al., 2021). The BC/MXene bioaerogels combine the excellent mechanical property of Ti_3_C_2_T_x_ MXene and many functional groups of BC, giving high pressure-sensitive and excellent ammonia-sensitive properties, revealing the necessity of the introduction of BC with 3D porous structures (as displayed in Figure 7). Moreover, the introduction of cellulose fibers allows the BC/MXene aerogel to degrade completely in a low-concentration H_2_O_2_ mixture, meeting the demands of sustainable development. Additionally, Kanno et al. utilized BC as a substrate to prolonged release of bone morphogenetic protein-2 (BMP-2) in a rabbit frontal sinus model for evaluating bone regeneration (Koike et al., 2019). The result suggests that BC can be used as a barrier membrane to maintain space and as a sustained-release drug carrier to promote bone formation. The aforementioned results show that BC-based composites have great potential in dental drug delivery.

### 3.4 Other BC-based drug delivery systems

In addition to the aforementioned application, BC-based biomaterials have sparked interest in intraperitoneal injection for drug delivery. Ishida et al. designed a novel paclitaxel (PTX) formulation for intraperitoneal injection via combining PTX with either carboxymethyl nano-fibrillated bacterial cellulose (CM-NFBC) or hydroxypropyl nano-fibrillated bacterial cellulose (HP-NFBC) for peritoneally disseminated cancers (Akagi et al., 2021). In the present research, NFBC was used as a substitute for Cremophor EL^®^ to reduce the side effects of PTX. Compared to albumin-bound PTX, the freeze-dried PTX/CM-NFBC and PTX/HP-NFBC are quickly restructured with preserved saline. An *in vivo* study indicated that the PTX formulations prolong the mean survival time in mouse models, as well as decrease systemic toxicity of free PTX, increasing the therapeutic efficacy. On the other hand, frequent bolus dosing, fluctuation of plasma drug concentrations, and drugs with short half-lives are necessary to be prepared as sustained release dosage forms (Ullah et al., 2016). Recently, some attempts have been made to manage the drug release in BC-based delivery systems. Wang et al. successfully prepared an aminoalkyl-grafted BC structure by alkoxysilane polycondensation employing 3-aminopropyltriethoxysilane (APTES) (He et al., 2020). Analysis results of morphology and chemical composition show that aminoalkylsilane groups were successfully grafted onto BC membranes through covalent bonding. Furthermore, the network of BC membranes is covered after grafting with APTES, resulting in decreased hydrophilicity. The antibacterial results demonstrate that aminoalkyl-grafted BC films have great potential in controlled drug delivery. Razak et al. reported the preparation of pH-sensitive composite hydrogels for wound-healing applications (Al-Arjan et al., 2022). BC is functionalized with different amounts of graphene oxide via a hydrothermal method and then cross-linked with polyvinyl alcohol using tetraethyl orthosilicate as a cross-linker using the blending method to fabricate composite hydrogels. The controlled drug release behaviors of BC-based hydrogels are studied at different pH levels, and the release kinetics are determined via various mathematical models. The results of swelling demonstrate that the composite hydrogels are extremely pH-sensitive, resulting in these BC-based hydrogels being suitable for controlled drug release, making them promising biomaterials for wound healing. Overall, BC has drawn great scientific attention in the past few decades. BC-based drug delivery carriers have been utilized for oral, transdermal, and dental drug delivery due to the unique structural and functional properties of BC. Although many studies present pH-responsive and controlled drug release profiles of BC-based drug delivery systems for long-term applications, a more sophisticated release behavior is still to be found. Furthermore, most of the research studies investigate the drug controlled release behavior in *in vitro* tests, while *in vivo* evaluation is still limited.

## 4 Conclusion and perspectives

Recently, BC has been a highly attractive candidate for the drug delivery system owing to its distinctive inherent properties such as good mechanical properties, high purity, microporosity, biocompatibility, and excellent water-holding capacity. However, it is hardly used in its pure form as drug delivery materials due to some inherent shortcomings of BC, including low activity, slow degradation, and restricted cell adhesion. Consequently, BC often needs to be modified to overcome these limitations, which ensures the biocompatibility and adjusts the hydrophilicity/hydrophobicity of BC to drug delivery. These modifications will affect not only the inherent properties of BC but also the sorption and release behavior of loading drugs. In this review, we also discussed the various routes of BC-based drug delivery, such as oral drug delivery, transdermal drug delivery, and dental drug delivery. In the case of the aforementioned drug delivery systems, the *in vivo* performance and bioavailability and *in vitro*/*in vivo* relation of the developed delivery systems need to be further studied.

Although researchers have presented promising results with the use of BC-based materials in drug delivery, most of the studies are still in the early stages. Some studies are just carried out in *in vitro* drug release, while others mainly focus on animal models. Therefore, further clinical trials need to be carried out to ensure the safety and efficacy of BC-based materials before being commercialized. Moreover, most drugs loaded in BC-based drug delivery systems are model drugs. More sophisticated drugs used for specific diseases are probably not suitable because of an unclear interaction between BC and different drugs. Additionally, the effect of various modifications on environmental and host physiological conditions is still not fully established, and therefore, advanced studies are needed as such drugs are loaded into BC. Nevertheless, several fascinating aspects suggested by authors are still worthy of being investigated further: 1) in order to improve drug delivery efficacy, optimization of BC-based composite formulation is needed; 2) some drugs, such as large-molecule drugs, drugs requiring a prolonged period to be delivered, and highly lipophilic drugs, applied in BC-based drug delivery need further investigation; 3) implementation in 3D printing for BC-based drug delivery methods require more attention as BC has high tensile strength and mechanical properties.

BC with modification will open a large range of applications in the biomedical field, such as artificial blood vessels, implant material, arterial stent coating, and wound dressing. Furthermore, BC can be a reliable material to develop high-technological diagnosis and treatment platforms. For instance, BC-based materials will improve the targeted ability of the drug delivery system and immobilize enzymes for *in vivo* treatment. Another application of BC-based materials is in portable electronic devices for smart e-skin, diagnostic sensors, and wound regeneration treatments. Due to its resemblance to human tissues, BC shows tremendous potential to be used in regenerative treatments and organ replacement with increase in the aging population. Despite many years of study in BC modification, there remains more to be discovered in biomedical application.
